# Combination of Enamel Matrix Derivatives with Bone Graft vs Bone Graft Alone in the Treatment of Periodontal Intrabony and Furcation Defects: A Systematic Review and Meta-Analysis

**DOI:** 10.3290/j.ohpd.b5871494

**Published:** 2024-12-05

**Authors:** Ibrahim Fidan, Julien Labreuche, Olivier Huck, Kevimy Agossa

**Affiliations:** a Ibrahim Fidan Postgraduate in Periodontics, University of Lille, Faculty of Dentistry, University Hospital of Lille, Department of Periodontology, Lille, France. Conceptualisation, study design, methodology, investigation, data collection, manuscript drafting.; b Julien Labreuche Biostatistics Engineer, University of Lille, University Hospital of Lille, Department of Biostatistics, EA 2694 – Public Health: Epidemiology and Quality of Care, Lille, France. Methodology, statistical analysis.; c Olivier Huck Professor, University of Strasbourg, Faculty of Dentistry Robert Franck, INSERM, UMR 1260, Regenerative Nanomedicine, Strasbourg, France. Methodology, investigation, data collection, manuscript review.; d Kevimy Agossa Professor, University of Lille, Faculty of Dentistry, University Hospital of Lille, Department of Periodontology, INSERM, U1008-Controlled Drug Delivery Systems and Biomaterials, Lille, France. Conceptualisation, project management, study design, methodology, investigation, data collection, manuscript review.

**Keywords:** bone substitutes, enamel matrix derivatives, furcation defects, intrabony defects, periodontal regeneration.

## Abstract

**Purpose:**

To compare the clinical performance of the combination of enamel matrix derivatives and bone substitutes (EMD+BG) with bone substitutes (BG) alone in the surgical treatment of periodontal intrabony and furcation defects.

**Materials and Methods:**

Electronic databases (Medline, Embase and Web of Science) were searched for randomised controlled trials in humans that investigated the combination of EMD+BG vs BG alone in either intrabony or furcation defects with a minimal follow-up of 6 months. A random-effect meta-analysis was conducted according to the type of defect (intrabony or furcation defects) and the follow-up time (6 or ≥ 12 months).

**Results:**

From a total of 1583 entries, 9 randomised controlled clinical trials (RCTs) were retrieved and included in the qualitative and quantitative synthesis. All of them were included in the meta-analysis. The meta-analysis detected an additional clinical attachment level (CAL) gain in intrabony defects treated with EMD+BG compared to BG alone in studies with ≥ 12-month follow-up (mean difference = 0.67 mm, 95% CI [0.44 ; 0.90], p < 0.00001). No additional benefit was found in furcation defects in terms of CAL gain or probing depth (PD) reduction.

**Conclusion:**

The addition of EMD may improve the clinical outcomes of intrabony defects treated with bone substitutes. These findings may support the use of this combined therapy, particularly in large and non-contained defects.

Periodontal intrabony and furcation defects are site-specific sequelae of the progression of clinical attachment loss and bone loss in periodontitis.^40^ Intrabony defects have been reported in 2%–18% of teeth, and furcation defects can affect up to 10% of molars.^29,30,36,38,69,70^ The prevalence of these defects increases with age and the severity of periodontitis.^29,36,38,69,70^ Both intrabony and furcation defects are associated with an increased risk of tooth loss in the absence of treatment.^35,37^ In a recent clinical guideline, it was recommended that, following completion of steps 1 and 2, teeth with residual deep pockets (>5 mm) associated with intrabony defects or class II furcation defects should be treated with periodontal regenerative surgery.^13,33,46^ Several reconstructive/regenerative strategies, including the use of membranes,^28,31^ bone substitutes (BG)^43^ or enamel matrix derivative (EMD)^6,7^ have been extensively investigated for the treatment of intrabony defects and furcation defects. While the majority of studies concluded that all these different reconstructive/regenerative therapies led to better outcomes than open flap debridement alone, systematic reviews have failed to identify which strategy would be most effective.^51^ In practice, it has been suggested that the choice of the biomaterial or possible combinations should be based on the defect configuration.^51,53,67^ Indeed, the morphology of the defect has long been considered to play a crucial role in clot stability and to influence the outcome of regenerative periodontal surgery. This hypothesis was thoroughly investigated in a recent meta-analysis by Nibali et al.^34^ They found that deeper defects were associated with greater radiographic bone gain (0.7 mm more for defects deeper than 4 mm compared to those 3-4 mm deep). Additionally, narrower angles were associated with increased bone and clinical attachment level (CAL) gain (approximately 1 mm more CAL gain for angles less than 37°), and more walls were associated with greater radiographic bone and CAL gain (approximately 0.5 mm more CAL gain per additional wall).^34^

Combination therapy refers to the simultaneous application of various periodontal reconstructive/regenerative strategies to obtain additive effects in comparison with monotherapies alone. This may be achieved by the assemblage of different reconstructive and regenerative materials to better address biological requirements of periodontal regeneration, including conductivity and inductivity, space provision and wound stability, matrix development, and cell differentiation.^32^ The combination of EMD+BG has been claimed as an attractive option for periodontal regeneration in large and non-contained defects.^11,13,33,46,52,56^ This relies on evidence that bone substitutes alone can serve as a scaffold but demonstrate inconsistent regenerative properties depending on the type of bone substitute employed.^11,43,51^ EMD contains signaling molecules with the potential to induce periodontal tissue regeneration and has been widely used clinically as a wound healing enhancer.^8,16,22,54,61,62^ However, it provides only a poor support to the flap stabilisation when used in its commercial viscous gel form resulting in limited performance in non-contained defects.^53,67^ From a biological perspective, the combination of EMD and BG can be considered as a tissue engineering strategy, involving the use of BG as a scaffold loaded with EMD as signaling molecules.^59^ The clinical benefit of the combination of EMD+BG is partly supported by multiple systematic reviews showing improvement in clinical attachment level (CAL) gain and probing depth (PD) reduction with the use of EMD+BG compared to EMD alone in intrabony defects.^17,33,56^ A recent meta-analysis demonstrated that the combination of EMD with other biomaterials may improve clinical attachment level (CAL) gain, bone gain, and probing depth (PD) reduction compared to EMD alone in intrabony defects. Interestingly, among the regenerative materials assessed, only BG (demineralised bovine bone mineral [DBBM] and hydroxyapatite + tricalcium phosphate [HA/βTCP]) showed superior performance when combined with EMD compared to EMD alone. DBBM was significant for CAL gain (mean difference = 0.90 mm, 95% CI [0.37 ; 1.43]) and PD reduction (mean difference = 0.40 mm, 95% CI [0.09 ; 0.71]), while HA/βTCP was significant only for bone gain (mean difference = 0.67 mm, 95% CI [0.40 ; 0.94 m]).^33^

However, the comparison of EMD+BG to BG alone has been poorly documented. Only a single systematic review focusing on intrabony defects has previously addressed this question and failed to support additional benefits of EMD as an adjuvant to BG.^64^ The findings of this systematic review were limited by the small number of studies which were available at the time. Additional clinical studies have addressed this question meanwhile, some of these studies investigated adjuvant effect of EMD on BG in furcation defects. Therefore, the aim of the present meta-analysis is to provide an up-to-date evaluation of the potential benefit of the combination therapy (EMD+BG) compared with bone substitutes alone (BG) in the treatment of intrabony and furcation defects.

## MATERIALS AND METHODS

### Protocol Registration and Reporting Format

The present review adheres to the Preferred Reporting Items for Systematic reviews and Meta–Analysis (PRISMA) statement.^25^ The study protocol was registered in the PROSPERO database (identification number CRD42023466749).

### Focused Question and Eligibility Criteria

The focused question was formulated as following: “Does the combination of EMD+BG provide additional clinical benefits compared with BG alone in terms of CAL gain, PD reduction, pocket closure, composite outcome of treatment success, gingival recession (REC) and bone gain in periodontal intrabony and furcation defects?”

Eligibility of studies was assessed using the Population, Intervention, Comparison, Outcomes, and Time (PICOT) framework as follows.^58^ Population (P): adult periodontitis patients (≥ 18 years old) with at least one intrabony or furcation defect; Intervention (I): periodontal regenerative/reconstructive surgical procedures involving the use of EMD combined with any type of bone substitutes (EMD+BG); Comparison (C): periodontal regenerative/reconstructive surgical procedures involving the use of bone substitutes alone (BG); Outcomes (O): Primary outcomes were CAL gain and PD reduction.

Secondary outcomes were percentage of pocket closure, composite measure of treatment success,^65^ soft-tissue wound healing, gingival recession (REC), tooth loss, patient-reported outcome measures (PROMs) and adverse events; Time (T): Minimum follow-up of 6 months following the surgical procedure.

Only randomised controlled clinical trials (RCTs) with a split-mouth or parallel design including at least 10 patients per arm were considered. RCTs with unclear/not specified type of treated intrabony or furcation defect were excluded. No time limitation was applied, and only articles published in English were considered after the electronic research.

### Information Sources and Search Strategy

The electronic databases Medline, Embase and Web of Science were searched up to July 2023 using a combination of MeSH terms and free-text words.

(1) Intervention: (‘enamel matrix derivatives’ OR ‘Emdogain’ OR ‘amelogenins’ OR ‘bone graft’ OR ‘bone substitute’ OR ‘graft’)
(2) Defects: (‘intrabony defect’ OR ‘periodontal defect’ OR ‘defect’ OR ‘furcation defect’ OR ‘interradicular defect’)
(3) Study: (‘randomised controlled trials’ OR ‘RCT’ OR ‘randomised clinical trials’)
(4) Combination: (1) AND (2) AND (3)

Additionally, a manual search was conducted in the major journals in the field: Journal of Periodontology, Journal of Clinical Periodontology, Journal of Periodontal Research, International Journal of Periodontics and Restorative Dentistry. Previous systematic reviews on the surgical treatment of intrabony and furcation defects were also screened for additional publications.^11,13,14,17,24,26,33,34,43,51,52,56,64^

### Article Selection Process 

Two reviewers (I.F. and K.A.) independently screened the titles and abstracts of all the entries identified in the literature search. The full text was searched for studies that were potentially eligible or for which the data contained in the abstract were insufficient to reach a decision. Any article considered potentially relevant by at least one of the reviewers was considered for full-text analysis. The full-text analysis was carried out independently by the same reviewers. Any disagreements were resolved by discussion or, in the absence of consensus, by consulting a third reviewer (O.H.). Articles that did not fulfill the eligibility criteria were excluded, and the reasons for exclusion were reported. In the case of missing data, a request was sent by e-mail to the authors.

### Data Extraction 

Data of the included articles were extracted using a standard extraction form specifically designed for this review. CAL change was the mean clinical attachment level increase or decrease in millimeters at follow-up visit. PD change was defined as the mean variation in periodontal probing depth in millimeters at follow-up visit. Pocket “closure” was defined as the presence of PD ≤ 4 mm without bleeding on probing (BOP) following the treatment. Treatment success was defined as the number or percentage of treated teeth that present a combination of “clinically relevant” CAL gain (≥ 3 mm) and pocket “closure” with PD ≤4 mm at study follow-up.^65^ REC change was the mean difference in recession height in millimeters between baseline and follow-up visits. Tooth loss was the number or the percentage of treated teeth that resulted missing (extracted) at the follow-up visit. PROMs and adverse events were collected under a narrative form when available. Additionally, the following study characteristics were extracted : (i) year of publication; (ii) design of the study (split-mouth vs parallel arm, single vs multicenter); (iii) characteristics of the population including age, gender, number of participants and treated sites (baseline/follow-up); (iv) characteristics of the intrabony defects (including number of remaining walls), horizontal and vertical classification of the furcation defects, and probing depth; (v) type of surgical procedure; (vi) biological agent and bone substitutes used; (vii) follow-up time points.

### Methodological Quality and Risk of Bias Assessment

The quality assessment was conducted independently by two reviewers using the Cochrane Collaboration’s tool (ROB-2)^9,57^ based on the assessment of five domains: randomisation process, deviations from the intended interventions, missing outcome data, measurement of the outcome and selection of the reported result. The risk of bias was assessed for each study as:

A. Low risk of bias if all criteria were met.
B. Unclear risk of bias if one or more criteria were partly met.
C. High risk of bias if one or more criteria were not met.

### Synthesis of the Evidence and Meta-Analysis

#### Qualitative synthesis

A narrative summary of the main characteristics and findings of the included studies was provided.

#### Quantitative synthesis

For the main outcomes of interest (CAL gain and PD reduction), the mean±standard deviation (SD) of baseline and follow-up measures (6- or 12-month follow-up) in each treatment group, and the mean±SD of change in measures from baseline to follow-up were extracted. When mean±SD were not reported in the text, they were estimated from figures or by using median (IQR) values assuming a normal distribution. When the SD of change was not provided and could not be estimated from IQR values, the SD of change was estimated using the average of SD of measures (baseline and last follow-up) assuming a correlation between repeated measures of 0.5. Mean differences between intervention and control groups in the change from baseline to follow-up measure (6-month or 12-month follow-up in cases of absence of 6-month evaluation) were calculated. Study heterogeneity in effect sizes was quantified using a homogeneity test based on Q statistics and by calculating the I² statistics; heterogeneity was interpreted by assessing the I² values as low, moderate, and high for I² values of 25%, 50%, and 75%, respectively. The pooled effect size for each outcome was estimated using the inverse variance-weighted method with fixed effect model or with the random-effect (DerSimonian and Laird) model in case of substantial heterogeneity (I² statistics > 50%). The analysis was done separately according to the type of defect (intrabony or furcation defects). Given the small number of studies, neither funnel plots for evaluation of publication bias nor meta-regression to explain heterogeneity could be provided. Statistical tests were conducted at the two tailed α-level of 0.05. Data were analysed using the Cochrane Collaboration’s Review Manager Software package (RevMan; edition 5.4).

## RESULTS

**Fig 1:**
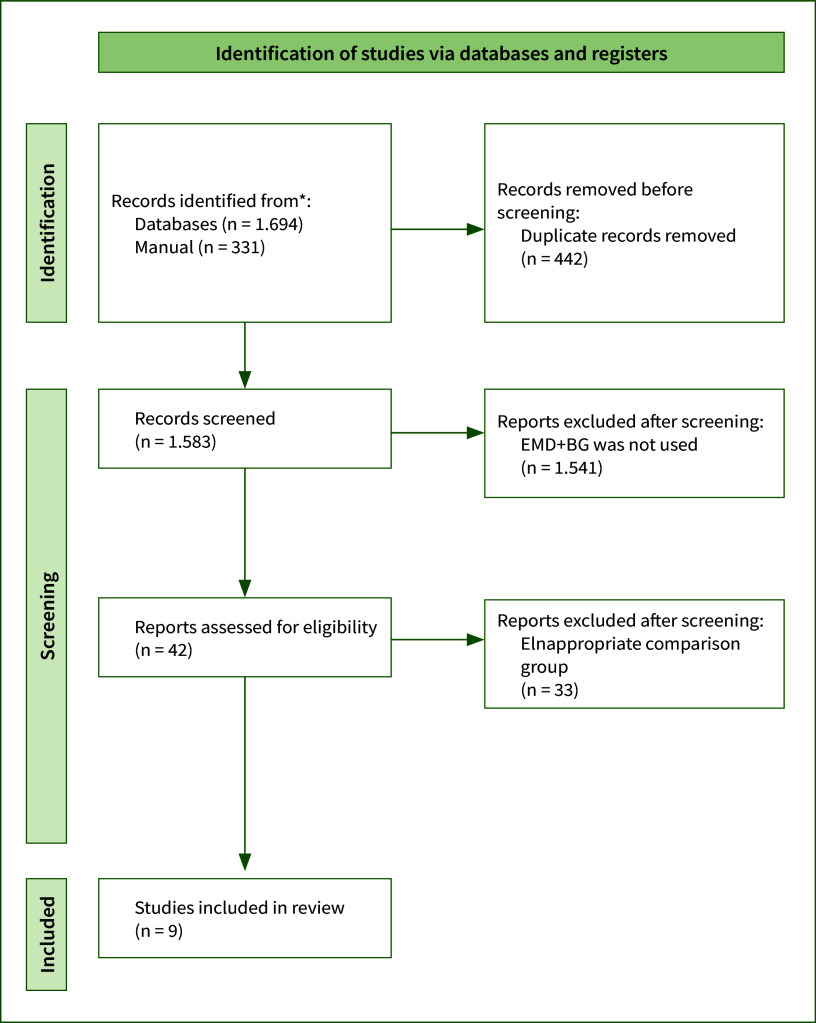
PRISMA flow diagram.

Figure 1 shows the literature review flow diagram. A total of 2025 articles were identified (1694 with the electronic search, 331 with the manual search). After duplicate removal, 1583 were screened by title and abstract and 42 reports were assessed for eligibility. Nine studies were finally included in the qualitative synthesis and meta-analysis.

### Study Characteristics

**Fig 2:**
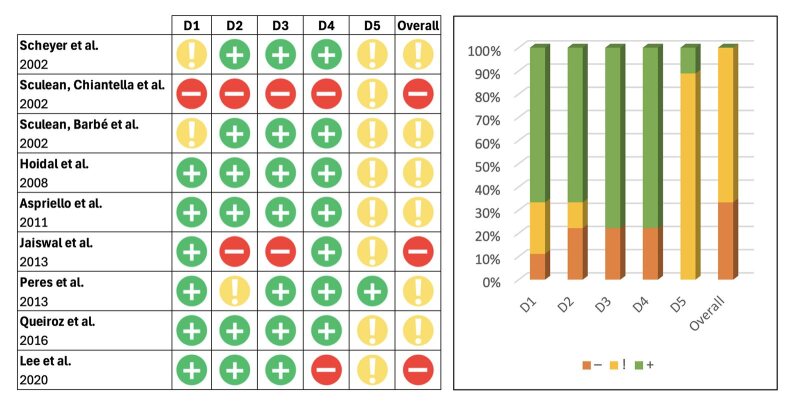
Risk of bias ROB 2.0 of included studies. D1: Randomisation process; D2: Deviations from the intended interventions; D3: Missing outcome data; D4: Measurement of the outcome; D5: Selection of the reported result.

Table 1 shows the main characteristics of the included studies. All the studies were RCTs (8 parallel arm and 1 split-mouth), with a follow-up of 6 to 24 months, published between 2002 and 2020. A total of 304 patients (221 intrabony defects and 88 furcation defects) were included. Four studies included smokers.^10,48–50^ Different types of bone substitutes were used including bovine- or porcine-derived xenografts,^15,48,50^ synthetic bioglass^39,42,49^ or allografts.^2,10,12^ Three studies had a high risk of bias^12,15,50^ and six presented an unclear risk of bias^2,10,39,42,48,49^ (Fig 2).

**Table 1:** Summary table of all studies included in the analysis

Reference	Study type	Follow-up	Lesion type	Bone substitute type	Test group	Control group	No. of patients	Smokers	No. of defects	Defect morphology
Test	Control	Test	Control	Test	Control
Scheyer et al, 2002	RCT Split- mouth	6 months	Intrabony defects	Xenograft	EMD+BDX	BDX	17	17	3*	–	17	17	Test: 14 2-3-wall 3 3-wall Control: 13 2-3-wall 4 3-wall
Sculean et al, 2002	RCT Parallel arm	12 months	Intrabony defects	Xenograft	EMD+BDX	BDX	12	12	2	3	12	12	Test: 2 1-wall 6 2-wall 4 3-wall Control: 2 1-wall 5 2-wall 5 3-wall
Sculean et al, 2002	RCT Parallel arm	12 months	Intrabony defects	Synthetic bioglass	EMD+BaG	BaG	14	14	4	3	14	14	Test: 3 1-2-wall 9 2-wall 2 3-wall Control: 4 1-2-wall 8 2-wall 2 3-wall
Hoidal et al, 2008	RCT Parallel arm	6 months	Intrabony defects	Allograft	EMD+DFDBA	DFDBA	32**	–	4	3	17	20	Test: 1 1-wall 1 2-wall 6 3-wall 9 combined Control: 2 1-wall 1 2-wall 6 3-wall 11 combined
Aspriello et al, 2011	RCT Parallel arm	12 months	Intrabony defects	Allograft	EMD+DFDBA	DFDBA	28	28	Smokers excluded	28	28	Test: 14 2-wall 14 3-wall Control: 13 2-wall 15 3-wall
Jaiswal et al, 2013	RCT Parallel arm	12 months	Furcation defects	Allograft	EMD+DFDBA	DFDBA	30***	–	Smokers excluded	30	–	Mandibular molars class II furcation defects
Peres et al, 2013	RCT Parallel arm	6 months	Furcation defects	Synthetic bioglass	EMD+βTCP/HA	βTCP/HA	15	15	Smokers excluded	Total defects: 30	Class II furcation defects
Queiroz et al, 2016	RCT Parallel arm	6 and 12 months	Furcation defects	Synthetic bioglass	EMD+βTCP/HA	βTCP/HA	14	14	Smokers excluded	14	14	Mandibular molars class II furcation defects
Lee et al, 2020	RCT Parallel arm	6 to 24 months	Intrabony defects	Xenograft	EMD+DPBM	DPBM	20	22	Smokers excluded	20	22	Test: 20 1-wall Control: 22 1-wall
RCT: randomised controlled trial; EMD: enamel matrix derivatives; BDX: bovine-derived bone xenograft; BaG: bioactive glass; DFDBA: demineralised freeze-dried bone allograft; βTCP/HA: β-tricalcium phosphate/hydroxyapatite; DPBM: deproteinised porcine bone mineral. *There were three smokers IN TOTAL in this study (not 3+3); unclear whether these 3 participants were in the test or control group. **32 patients in total (test and control combined). No breakdown by group. *** 30 patients total (test and control combined).

### Clinical Attachment Level Gain

**Fig 3:**
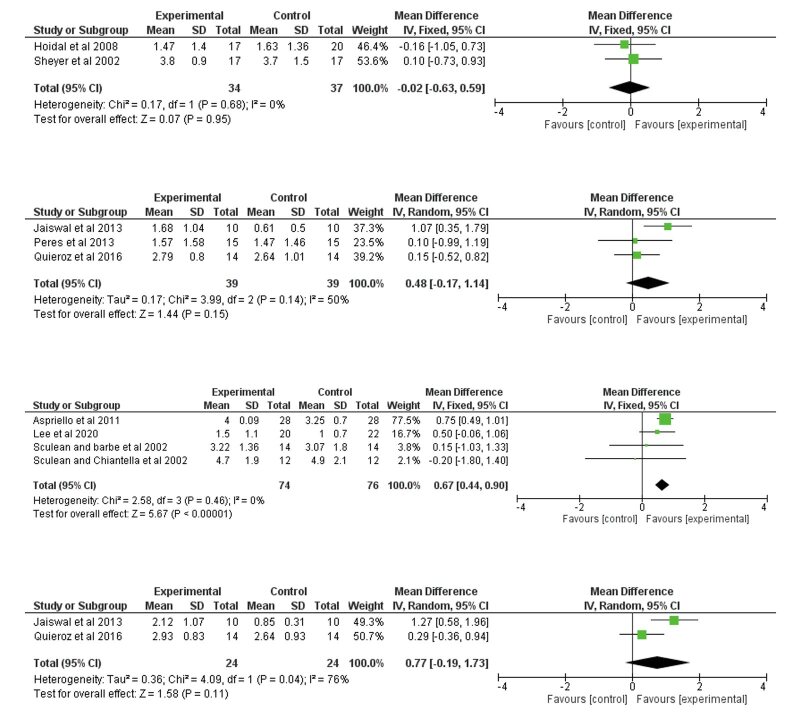
Meta-analysis assessing the benefits of combined therapy for clinical attachment level gain in the regeneration of periodontal defects. 3a. Clinical attachment level gain in intrabony defects at 6 months; 3b. Clinical attachment level gain in furcation defects at 6 months; 3c. Clinical attachment level gain in intrabony defects at 12 months; 3d. Clinical attachment level gain in furcation defects at 12 months.

At 6 months, no difference was detected between EMD+BG and BG alone groups for clinical attachment level (CAL) gain in either intrabony defects^10,48^ (Fig 3a) or furcation defects^12,39,42^ (Fig 3b). However, the combination of EMD+BG yielded an additional CAL gain in intrabony defects at 12 months^2,15,49,50^ (mean difference = 0.67 mm, 95% CI [0.44 ; 0.90], p < 0.00001) (Fig 3c), but no difference was found in furcation defects^12,42^ (Fig 3d).

### Probing Depth Reduction

**Fig 4:**
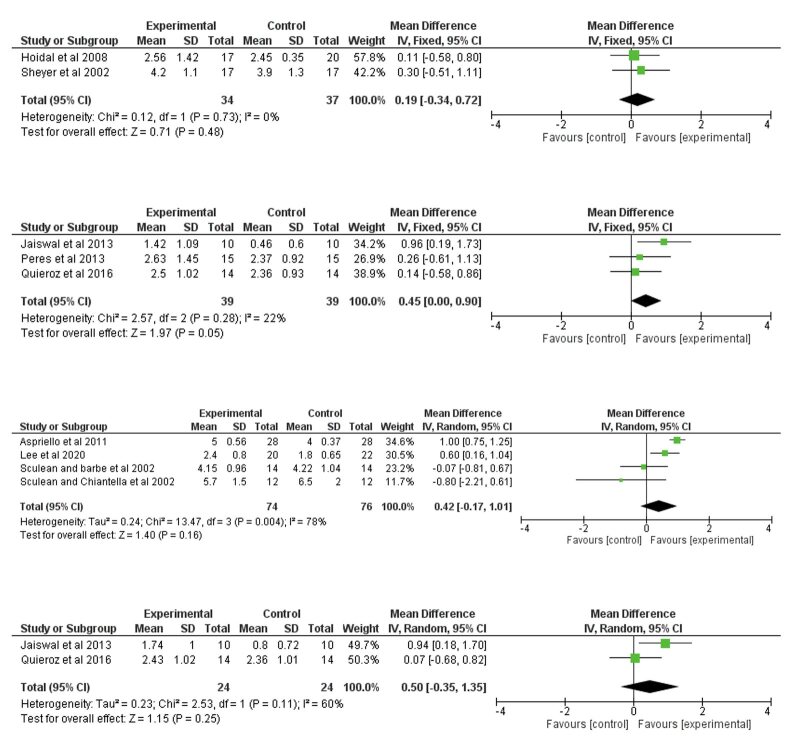
Meta-analysis assessing the benefits of combined therapy for probing depth reduction in the regeneration of periodontal defects. 4a. Probing depth reduction in intrabony defects at 6 months; 4b. Probing depth reduction in furcation defects at 6 months; 4c. Probing depth reduction in intrabony defects at 12 months; 4d. Probing depth reduction in furcation defects at 12 months.

Based on 6-month follow-up studies, no statistically significant difference was detected for PD reduction in intrabony defects^10,48^ (Fig 4a). For furcation defects, a mean difference of 0.45 mm (95% CI [0.00 ; 0.90], p = 0.05) was measured^12,39,42^ for PD reduction at 6 months (Fig 4b). Based on 12-month follow-up studies, the meta-analysis did not detect any difference between EMD+BG or BG alone groups for PD reduction either in intrabony defects^2,15,49,50^ (Fig 4c) or furcation defects^12,42^ (Fig 4d).

Overall, in 6-month follow-up studies, a low to moderate level of heterogeneity was detected for PD reduction (I² = 0%, p = 0.73; I² = 22%, p = 0.28) and CAL gain (I² = 0%, p = 0.68; I² = 50%, p = 0.14). In 12-month follow-up studies, the level of heterogeneity was low to high for PD reduction (I² = 78%, p = 0.004; I² = 60%, p = 0.11) and CAL gain (I² = 0%, p = 0.46; I² = 76%, p = 0.04).

No study reported tooth loss or success rates based on composite endpoints. However, the number of closed pockets after treatment could be retrieved for only one study.^15^ In that study, which focused on 1-wall intrabony defects, the authors indicated that approximately 5% of the sites treated with BG alone achieved a “closed pocket” (PD≤4 mm) (1/22 sites at 2 years and 1/18 sites at 4 years), whereas no closed pocket was achieved in the EMD+BG group. The difference was not statistically significant.

### PROMS and Adverse Events

Only one study evaluated PROMs and reported less pain intensity (p = 0.046), duration (p = 0.033), and swelling (p = 0.022) in the EMD+BG group compared to BG alone.^15^ In the same study, minor adverse events occurred, including dehiscence and/or fenestration, persistent swelling, spontaneous bleeding and ulceration in 5%, 10%, 5% and 10% of treated sites in the EMD+BG group compared to 18.2%, 27.3%, 9.1% and 4.5% of sites treated with BG alone. The difference was not statistically significant.

## DISCUSSION

Combination therapy refers to the simultaneous application of various periodontal reconstructive/regenerative strategies to obtain additive effects^32^ in comparison with monotherapies alone. Indeed, they combine both mechanical and biological properties of selected materials to achieve periodontal reconstruction and CAL gain, especially in wide and deep defects where wound stability is more challenging to obtain. Systematic reviews have documented improved outcomes with EMD+BG compared to EMD alone, but there was a controversial issue regarding the potential benefit of the combination therapy compared to BG alone.^64^ To address this question, this systematic review identified nine randomised controlled trials including a total of 309 periodontal intrabony or furcation defects in 304 patients who received either EMD+BG as a combination or BG as a monotherapy. The clinical outcomes were reported at short- (6 months) and medium-term (≥12 months) based on the typical follow-up durations in most of the clinical studies on this topic.

### Main Findings

Overall, our meta-analysis detected an additional CAL gain in intrabony defects treated with EMD+BG compared to BG alone in studies with ≥12-month follow-up. This adjunctive clinical effect could not be confirmed in furcation defects, even if the mean difference for PD reduction was almost statistical significance at 6 months, in favour of the EMD+BG group. The positive results observed in intrabony defects support data showing that EMD may further improve the outcomes of periodontal regenerative surgery by promoting and accelerating wound healing and periodontal regeneration, and reducing the risk of postoperative complications, leading to better clinical outcomes.^23,44,47,63,68^ Interestingly, the findings of one study might suggest improved early wound healing, in terms of reduced flap dehiscence or fenestration and persistent swelling, in the EMD+BG group compared to BG alone, but the difference was not statistically significant.^15^ In vitro, the addition of EMD to various BG has shown positive effects on cell adhesion, proliferation and differentiation, as well as the regulation of biological mechanisms involved in tissue healing, which could explain these findings.^18–21,27^ To provide further support for the use of BG in combination with a biological agent, it has also been suggested that BG alone would be less likely to promote a “true” periodontal regeneration due to the encapsulation of the particles in the connective tissue.^51^ However, it is important to keep in mind that even if EMD is considered a well-documented pro-regenerative agent, histological evidence of periodontal regeneration has been reported in less than 50% of intrabony defects treated with EMD.^51^ Furthermore, the extent to which “true” regeneration leads to better results in terms of long-term periodontal stability and tooth retention has not been demonstrated.

### Comparison with Current Literature

Our results are in line with a recent systematic review and network meta-analysis assessing the effect of a large set of biologics including autogenous blood-derived products (including platelet-rich plasma, PRP and platelet-rich fibrin, PRF), EMD and growth factors (rh-PDGF-BB), that showed that the addition of biologic agents to BG may improve the clinical and radiographic outcomes, as compared to BG and flap procedures alone.^60^ Early reviews have failed to support additional clinical benefits of EMD compared to BG in the treatment of intrabony defects.^24,64^ It should be noted that Troiano et al^64^ included only 5 RCTs and Miron et al^24^ did not perform a meta-analysis. The inclusion of more studies may have enabled us to detect some statistically significant inter-group differences. Surprisingly, EMD+BG resulted in additional improvements in terms of CAL gain compared to the BG alone group, but the difference was not statistically significant for probing-depth reduction. Similar findings were reported by Matarasso et al^17^ when comparing EMD+BG to EMD alone. The explanation of this finding is unclear. It could only be speculated that the improved CAL gain in the EMD+BG group may be related to a reduced postoperative gingival recession. Indeed, it has been shown that EMD application in root coverage surgeries resulted in better outcomes in terms of gingival recession coverage, as well as a statistically significant increase in vascular endothelial growth factor (VEGF) expression, suggesting that EMD may enhance the soft-tissue angiogenic and healing process.^5^ Due to the lack of available data on postoperative gingival recession in the majority of the selected studies, a meta-analysis could not be conducted to confirm this hypothesis.

An important finding of this systematic review is the lack of additional benefit of EMD in class II furcation defects treated with BG. This finding is consistent with the conclusions of two previous meta-analyses.^13,55^ Soares et al^55^ found no statistically significant difference in any of the outcomes when comparing EMD + HA/βTCP to HA/βTCP alone and concluded that adding EMD to other materials may not be beneficial in class II furcation defects. Jepsen et al^13^ conducted a Bayesian network meta-analysis, showing that BG had the highest probability of being the most effective treatment compared to the other regenerative strategies. The reasons for the lack of effect at these specific sites remain speculative. It has been suggested that the microbiome and molecular signature of furcation defects differ considerably from interproximal sites, which might indicate that furcation anatomy could lead to unique environmental characteristics affecting microbial diversity and host response.^41,45^ One study demonstrated the antimicrobial effect of EMD, used alone or in combination with BG, in class II furcation defects.^41^ However, the extent to which this effect is comparable to intrabony defects remains unknown and should be addressed in future research.

### Limitations 

The small number of included studies and their limited follow-up (only one study reported follow-up beyond one year) are obvious limitations of this systematic review. Additionally, the studies were highly heterogeneous regarding the morphology of intrabony defects. One study focused exclusively on one-wall defects,^15^ two studies combined data from two-, three-, and two-to-three-wall defects,^2,48^ and three studies included defects with one, two, and three walls or combined defects.^10,49,50^ Similarly, there was very limited data on flap design. The only details provided related to mucoperiosteal flaps, with or without releasing incisions, and some studies merely noted that interdental tissues were preserved. Therefore, when interpreting the present results, it should be noted that the meta-analysis combined data from studies using different flap designs with heterogeneous morphology of intrabony defects, treated with various bone substitutes. Due to the lack of data, we were unable to perform a subgroup analysis based on these factors, which have been reported to affect the outcomes of regenerative treatments.^1,3,4,34,51,66^ Another limitation was the inability to compare radiographic bone-fill outcomes, as only a few studies provided this outcome and it has been differently measured across studies.

## CONCLUSION

The addition of EMD may improve clinical outcomes of intrabony defects treated with bone substitutes. These findings may support the use of this combination therapy in large and non-contained intrabony defects, but no statistically significant benefit was detected in furcation defects.

Further studies are needed to support these conclusions and help to determine most effective treatment strategies while considering clinical outcomes and cost-benefit ratio.
